# Male reproductive system of the deep-sea acorn worm *Quatuoralisia malakhovi* (Hemichordata, Enteropneusta, Torquaratoridae) from the Bering Sea

**DOI:** 10.1186/s12983-024-00548-w

**Published:** 2024-10-08

**Authors:** Anastasiya Ivanovna Lukinykh, Olga Vladimirovna Ezhova, Vladimir Vladimirovich Yushin, Sergey Vladimirovich Galkin, Vladimir Vasilievich Malakhov

**Affiliations:** 1 Biological Faculty, Department of Invertebrate Zoology, Lomonosov Moscow State University, Leninskie Gory, 1, Bld. 12, Moscow, Russia 119234; 2 A.V. Zhirmunsky National Scientific Center of Marine Biology, Far Eastern Branch, Russian Academy of Sciences (NSCMB FEB RAS), Palchevskogo Str. 17, Vladivostok, Russia 690041; 3 Shirshov Institute of Oceanology, Russian Academy of Sciences, Nakhimovskiy Prospect, 36, Moscow, Russia 117997

**Keywords:** Gonad, Testis, Germinative epithelium, Spermatogenesis, Spermatozoa, Interstitial cells, Muscle cells, Gonad pore, Spawning, Bering sea

## Abstract

**Background:**

The deep-sea acorn worm *Quatuoralisia malakhovi* belongs to the phylum Hemichordata, class Enteropneusta, family Torquaratoridae, which was described in 2005. Owing to their epibenthic lifestyle and deep-sea habitat features, torquaratorids differ anatomically from shallow-water acorn worms; however, their morphology and fine structure are poorly studied. We have the opportunity to make three complete detailed series of histological sections of *Q. malakhovi* and to study the microscopic anatomy, histology and fine structure of the reproductive system of this acorn worm using scanning and transmission electron microscopy.

**Results:**

The sexes of *Q. malakhovi* are separate and indistinguishable externally. The lobed testes occupy the dorsal side of the genital wings and distinctly bulge into the peribranchial cavity by their mature lobes. The central part of the testis is always submerged into the genital wing and opens via a single gonad pore. The monociliary muscle cells stretch along the external wall of the testis and surround the gonad pore, probably taking part in the contraction of the testis lobes for spawning. The germinative epithelium of the testis contains spermatogenic cells at different stages of development and interstitial cells. Yolk cells are not found. Interstitial cells embrace the spermatogonia and spermatogenic columns, providing horizontal compartmentalization of the germinative epithelium, and contain numerous phagosomes with remnants of degenerating spermatogenic cells. The testis wall contains haemal lacunae, which are usually located on the side opposite the gonad pore. We describe the fine structure of spermatogonia, spermatocytes clustered in spermatogenic columns, spermatids, and spermatozoa. Spermatozoa are of the ectaquasperm type and consist of an acorn-shaped head and a flagellum 18–25 µm long. The sperm head includes a beak-shaped acrosomal part, a spherical nucleus and a midpiece containing a ring of 5 or rarely 6 mitochondria.

**Conclusions:**

The male reproductive system and sperm structure of *Q. malakhovi*, a representative of the family Torquaratoridae, have a number of differences from shallow-water acorn worms; however, the spermatogenesis and sperm structure of *Q. malakhovi* generally follow the pattern of the other three enteropneust families, and the phylogenetic significance of these deviations should be the subject of further research.

## Introduction

Hemichordata are marine invertebrates with traits similar to those of echinoderms and chordate animals. The Hemichordata phylum includes two classes: the sedentary colonial pterobranchs (Graptolithoidea) and solitary worm-like acorn worms (Enteropneusta). The class Enteropneusta currently includes four families: Ptychoderidae Spengel, 1893, Spengelidae Willey, 1899, Harrimaniidae Spengel, 1902 and Torquaratoridae Holland et al., 2005. Most species in the first three families live in shallow waters. The recently established family Torquaratoridae [1] is limited to the deep-sea and is updated with new findings almost every year. Unlike shallow-water enteropneusts, torquaratorids are epibenthic surface-dwellers that slowly crawl on the sea floor. Due to this lifestyle, they have often been found in the frames of deep-sea photo and video shooting in recent decades [2–5]. Eleven species of torquaratorids have already been described [1, 5–11], and according to the photo and video data, many torquaratorids still remain undescribed [2–5, 12]. Owing to their epibenthic lifestyle and deep-sea habitat, torquaratorids differ anatomically from shallow-water acorn worms [1, 5–11]. These differences affect the torquaratorid reproductive system. The family Torquaratoridae includes the only currently known hermaphrodite enteropneusts *Yoda purpurata* Priede, Osborn, Gebruk, Jones, Shale, Rogacheva & Holland, 2012 and *Y. demiankoopi* Holland, Hiley & Rouse, 2022 [8, 11]. Among enteropneusts only in representatives of the Torquaratoridae family, the external ovaries (in *Allapasus aurantiacus* Holland, Kuhnz & Osborn, 2012 and *A. fuscus* Jabr, Archambault & Cameron, 2018) and “external brooding” of the embryos within the ectodermal epithelium (in *Coleodesmium karaensis* Osborn, Gebruk, Rogacheva & Holland, 2013) were found [7, 9, 10]. However, there are currently no detailed morphological descriptions of the torquaratorid reproductive system.

**Fig. 1:**
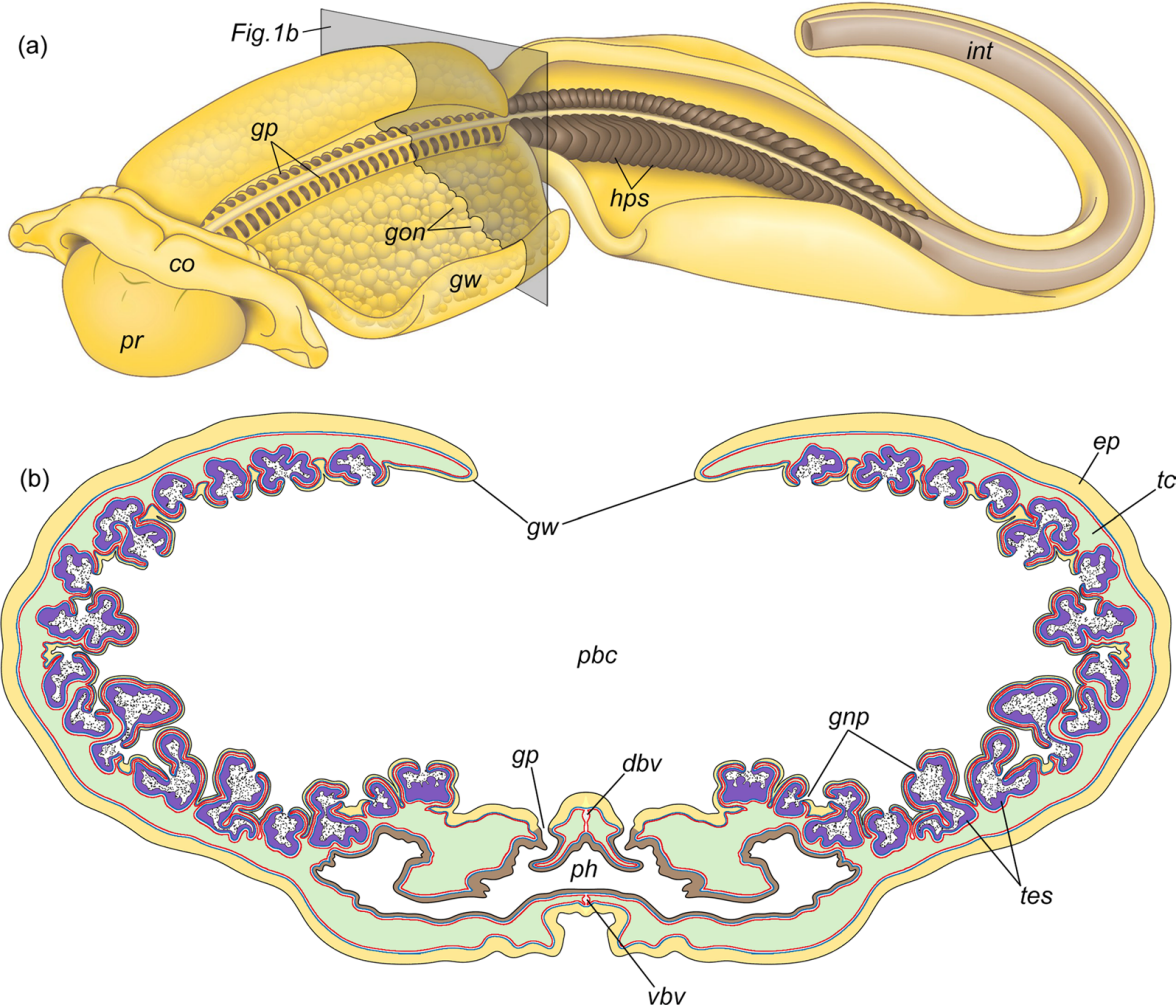
**a** Location of the gonads in the body of *Quatuoralisia malakhovi* and the level of the transverse section diagram. **b** Diagram of the transverse section through the branchiogenital subregion of the trunk. *co*, collar; *dbv*, dorsal blood vessel; *ep*, ectodermal epithelium; *gnp*, gonad pores; *gon*, gonads; *gp*, gill pores; *gw*, genital region of the lateral wing; *hps*, hepatic sacculations; *int*, intestine; *pbc*, peribranchial cavity; *ph*, pharyngeal cavity; *pr*, proboscis; *tes*, testes; *tc*, trunk coelom; *vbv*, ventral blood vessel

**Fig. 2:**
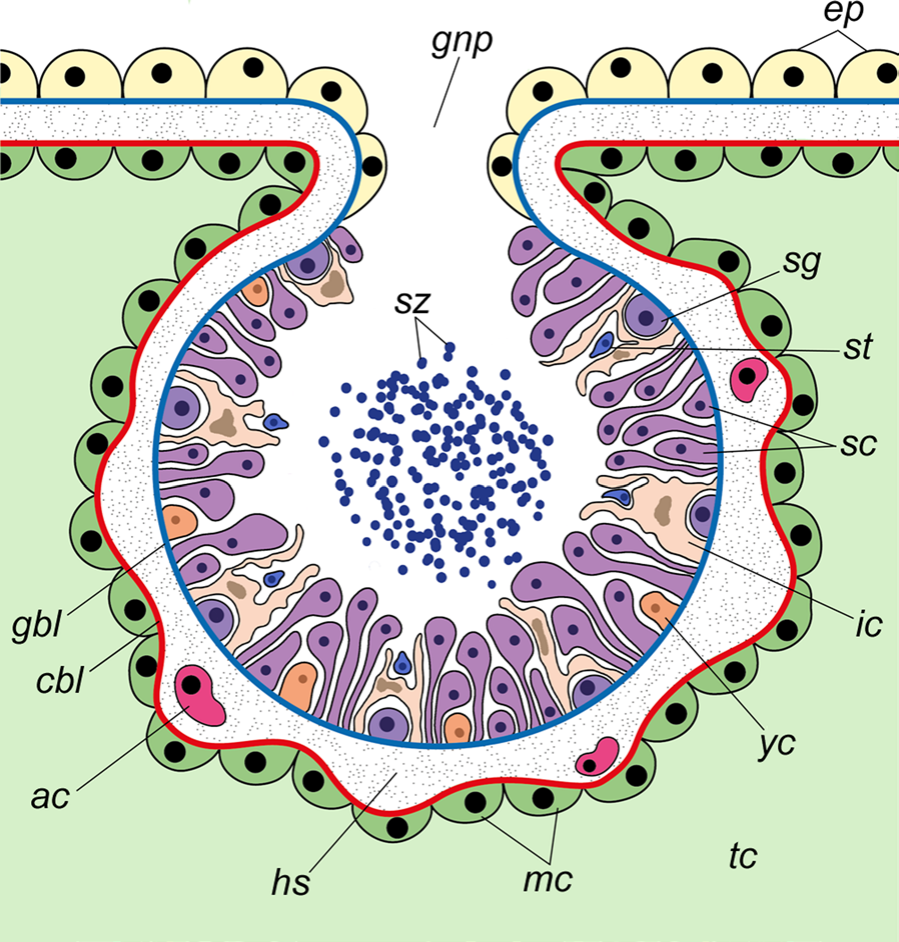
Generalized diagram of the testis of acorn worms; based on [14, 22–25]. *ac*, amoebocyte; *cbl*, coelothelial basal lamina (outer); *ep*, ectodermal epithelium; *gbl*, basal lamina of the germinative epithelium (inner); *gnp*, gonad pore; *hs*, haemal sinus; *ic*, interstitial cell; *mc*, muscle cell; *sc*, spermatocyte; *sg*, spermatogonium; *st*, spermatids; *sz*, spermatozoa; *tc*, trunk coelom; *yc*, yolk cell

The gonads of acorn worms develop in the trunk coeloms (left and right metacoels) and usually occupy either the lateral (= genital) wings (in most ptychoderids and torquaratorids—Fig. 1a, *gon*, *gw*) or the dorsolateral/lateral (= genital) ridges (in Harrimaniidae, Spengelidae, and some Ptychoderidae and Torquaratoridae) [1, 5, 7–11, 13–21]. Gonads of acorn worms are the elongated or lobed sacs, the inner wall of which is lined with germinative epithelium, and the outer wall consists of the muscle cells of the trunk coelom (Fig. 2). Thus, the gonad wall usually includes two basal laminae, the inner one belongs to the germinative epithelium (Fig. 2, *gbl*), and the outer one belongs to the trunk coelothelium (Fig. 2, *cbl*) [14, 22–25]. The remains of the primary cavity (haemocoel, connective tissue matrix) are located between these two basal laminae (Fig. 2, *hs*). The germinative epithelium of the testes includes spermatogenic cells and two types of somatic cells, namely, yolk cells and interstitial cells (Fig. 2, *yc*, *ic*). Yolk cells (if present) are usually found in both sexes, whereas interstitial cells are characteristic of only males. Interstitial cells form numerous slender processes, which envelop the germinal cells and delimit different stages of spermatogenesis [7, 13, 23, 24, 26]. Spermatogenic cells include **a** spermatogonia (Fig. 2, *sg*), large germ stem cells attached to the basal lamina; **b** spermatocytes (Fig. 2, *sc*), which are grouped into clusters elongated from the walls of the testis to its lumen; and **c** spermatids (Fig. 2, *st*), which throw the residual bodies and turn into **d** spermatozoa (Fig. 2, *sz*). All these cells are stratified within the testes in a radial direction, i.e., spermatogonia, spermatocytes, spermatids, and spermatozoa are arranged consecutively from the wall to the center of the testis. Additionally, spermatogonia and spermatocytes are often located in clusters separated from each other along the wall of the testis (Fig. 2). Thus, the different stages of spermatogenesis are spatially separate [22, 24, 27, 28]. Mature gonads open via gonad pores on the dorsal side of the trunk (Figs. 1b, 2, *gnp*). If the acorn worms have genital wings, the gonad pores open into the peribranchial cavity, which is formed by the arched wings (Fig. 1b, *pbc*) [7, 8]. It is assumed that the gonoducts and gonad pores, through which the mature gonads communicate with the environment, correspond to modified coelomoducts [14].

During cruises 75 (2016) and 82 (2018) of the research vessel *Akademik M.A. Lavrentyev* to the Bering Sea, an unusually abundant population of deep-sea acorn worms was found on the slopes of Piip Volcano (Volcanologists Massif) [29, 30]. A total of 16 specimens of these torquaratorids were collected, and later described as *Quatuoralisia malakhovi* Ezhova et Lukinykh, 2022. Thus, we have the opportunity to make a complete detailed series of histological sections of this acorn worm and to study its organs and tissues using scanning electron microscopy (SEM) and transmission electron microscopy (TEM). The description of the general anatomy of *Q. malakhovi* [5] includes a brief report of its reproductive system. Here, we provide the results of a detailed study of the microscopic anatomy, histology and fine structure of the male reproductive system of *Q. malakhovi* together with ultrastructural observations of testes with different stages of germ cells development, including mature spermatozoa.

## Material and methods

### Sampling and fixation

The studied specimens of *Q. malakhovi* (Table 1) were collected during the 75 (June 27, 2016), and 82 (June 18, 2018) cruises of the RV *Akademik M.A. Lavrentyev* to the Bering Sea at depths of 2289 m (55.4609N; 167.2688E) and 1957–1933 m (55.3451–55.3466N; 167.2750–167.2752E) respectively using a slurp-gun of the Comanche 18 remotely operated underwater vehicle (ROV). The freshly collected specimens were photographed using a Nikon D800 camera with a Micro Nikkor 60 macro lens and a Nikon SB600 off-camera flash (the photos were taken by Nadezhda Sanamyan). All specimens were fixed in 8% formaldehyde in seawater; the samples were stored in this fixative for one year in a refrigerator (–4 °C) before further processing.

**Table 1:** Material studied and methods used

Specimen/Sample no	Station	Studied material	Preservation	ID (if available)	Collection /Museum no	Method used	Reference to figures
1	LV 82–9	Whole specimen	–	1 (1375)	–	External study	3a
2	LV 75–17	Branchiogenital subregion of the trunk	8% formaldehyde in sea water	–	2017-QM-01	Histology (7 μm transverse sections)	–
3	LV 75–17	Fragment of genital wing	8% formaldehyde in sea water	–	2018-QM-03	External study; histology (10 μm sections); SEM	3b-d, 4e, 5c
4	LV 82–9	Fragment of genital wing	8% formaldehyde in sea water	4 (1378)	2020-QM-05	Histology (10 μm sections); 3D reconstruction;SEM	4a-d, 5a,b,d-h, 13a
5	LV 82–9	Fragment of genital wing	8% formaldehyde in sea water	(1382)	–	TEM, SEM	6, 7b,c, 8–12, 13b–f

### External morphology and histological study

The external morphology was studied using photographs of freshly collected specimens, and preserved material (Table 1, nos. 1 and 3). Photographs of the preserved material were taken using a Leica M165C stereomicroscope with a Leica DFC490 camera (Leica Biosystems, Germany) and an MSP-2 var.2 stereomicroscope with an MC-12 digital camera (LOMO, Saint-Petersburg, Russia).

Prior to histological processing, selected fragments of the genital wings were removed, dehydrated through increasing series of ethanol and butanol, embedded in paraplast, and sectioned into 7 μm and 10 μm thick sections using a rotational microtome Leica RM 2125RTS (Leica Biosystems, Germany). Sections were stained with hematoxylin and eosin following standard procedures [31]. In total, three series were studied using light microscopy (Table 1, nos. 2–4). Photographs of the histological sections were obtained using a Micmed-6 microscope (LOMO, Saint-Petersburg, Russia) with an MC-12 digital camera. The studied material is deposited in the collection of the Student Laboratory of Evolutionary Morphology of Animals (www.evolmorphan.ru), Department of Invertebrate Zoology, Biological Faculty of Lomonosov Moscow State University.

A series of histological sections of specimen no. 4 (Table 1) was used for 3D reconstruction of the testis. The reconstruction was carried out using AMIRA software, version 6.5.0. In total, 133 slides were used for reconstruction.

### Scanning electron microscopy (SEM)

The samples of genital wings for SEM (Table 1, no. 3–5) were dehydrated through acetone following standard procedures [32], critical-point dried using CO_2_ (HCP-2 Critical Point Dryer, Hitachi, Japan), sputter-coated with a gold–palladium mixture (EIKO IB-3 Ion Coater, Giko Engineering, Japan) and examined using SEM JSM-6380LA (JEOL Ltd., Japan) and Camscan-S2 (Cambridge Instruments, UK).

### Transmission electron microscopy (TEM)

The samples for TEM (parts of genital wings containing testes, Table 1, no. 5) were additionally fixed in a 2.5% glutaraldehyde solution buffered with 0.1 M sodium cacodylate (pH 7.2–7.4). Postfixation was performed in 1% osmium tetroxide buffered with sodium cacodylate (pH 7.2–7.4). The samples were subsequently dehydrated through an increasing series of ethanol and acetone following standard procedures [32], embedded in Epon epoxy resin and sectioned into 70–80 nm thick sections. These ultrathin sections were contrasted with uranyl acetate and lead citrate. The sections were examined using a JEOL JEM-1011 TEM with an ORIUS SC1000W digital camera (JEOL Ltd., Japan) and a JEOL JEM-1400 Flash TEM (JEOL Ltd., Japan).

## Results

### General morphology of the male reproductive system

**Fig. 3:**
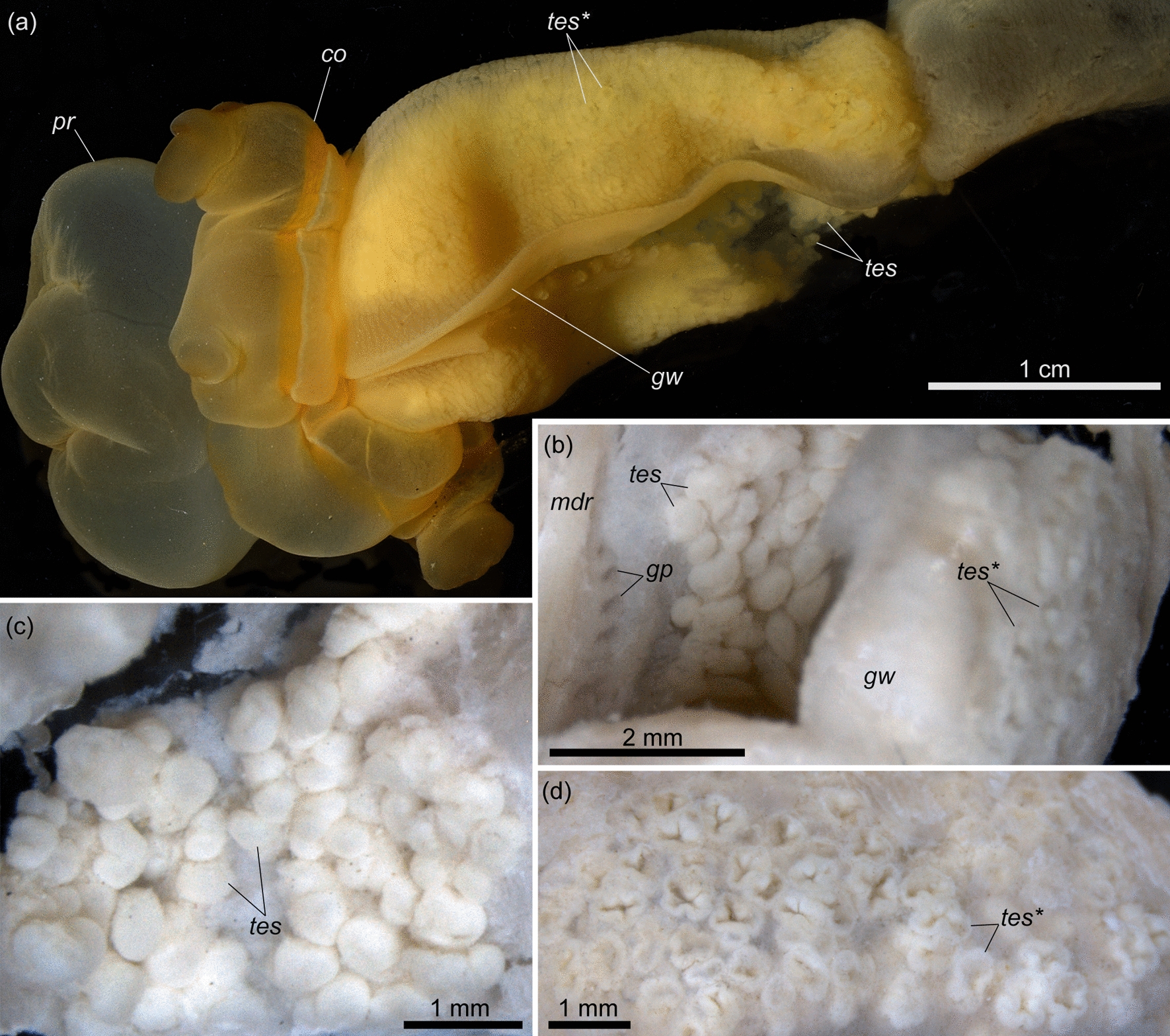
External view of the testes of *Quatuoralisia malakhovi*. **a** Testes in the branchiogenital subregion of the trunk in the freshly collected specimen. **b** The peribranchial cavity with gill pores (*gp*) and protruding testes (*tes*) and the external side of the genital wing with the testes visible through the semitranslucent ectodermal epithelium (tes*). **c** Testes protruding into the peribranchial cavity. **d** Testes in the form of corrugated funnels. *co*, collar; *gp*, gill pores; *gw*, genital wing; *mdr*, middorsal ridge; *pr*, proboscis; *tes*, testes; tes*, testes visible through a semitranslucent genital wing

*Q. malakhovi* is a gonochoristic species without sexual dimorphism; females and males are indistinguishable by external morphology. Gonads are visible through the semitransparent trunk and are of white, light yellow, cream, or light brown color. Testes occupy the coelom of wide genital wings in the whole branchiogenital subregion (Fig. 3a) from the collar/trunk septum to the hepatic subregion, sometimes slightly entering the latter. The testes are located on the dorsal side of the genital wings (Fig. 1b) and protrude distinctly into the peribranchial cavity (Fig. 3b, c, *tes*). On the opposite side of the wing, the testes of the fixed specimens are translucent in the form of corrugated funnels (Fig. 3d, tes*).

**Fig. 4:**
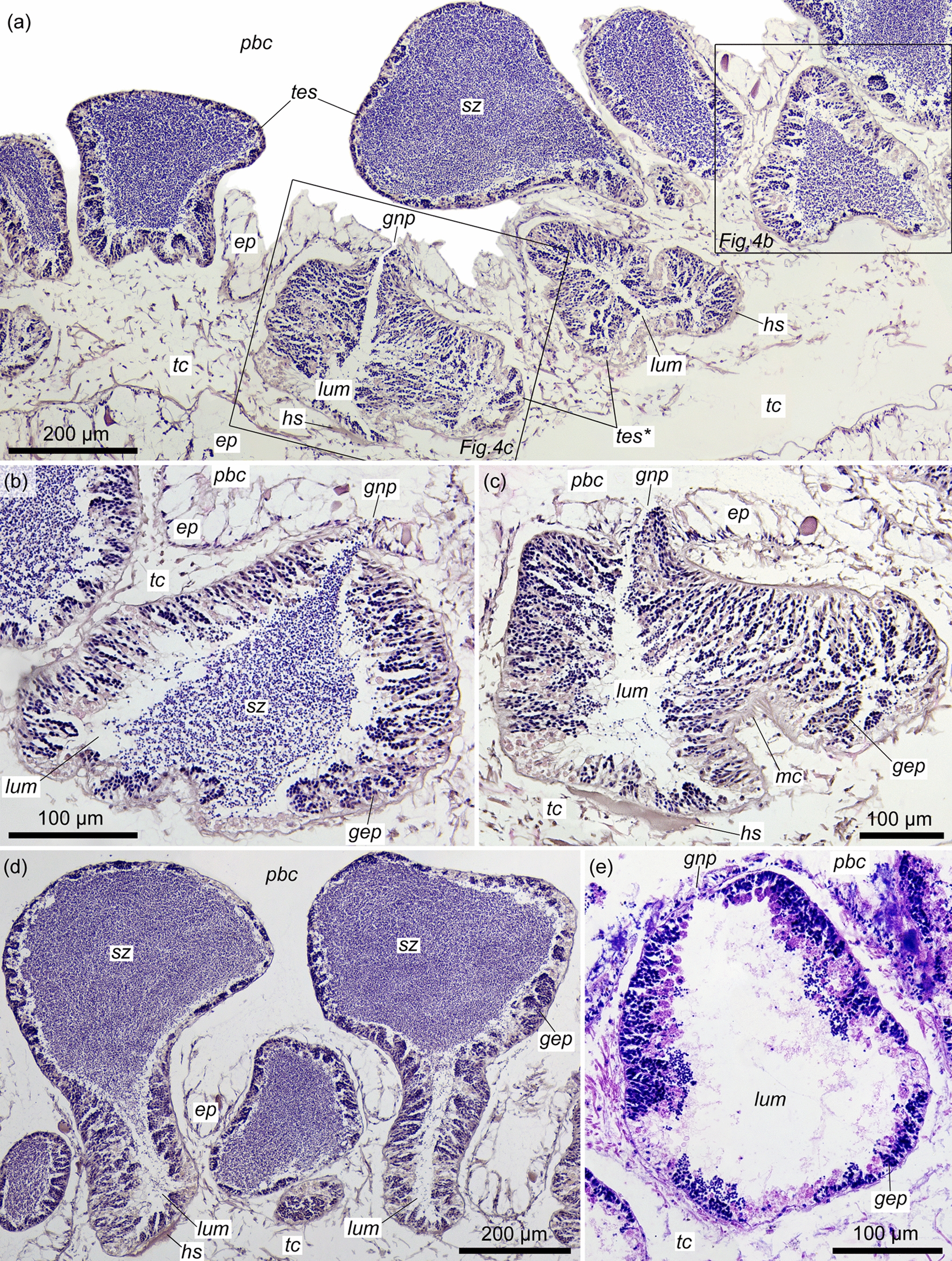
Histological structure of the testes of *Quatuoralisia malakhovi*. **a** Section of the genital wing of male *Quatuoralisia malakhovi* (frames indicate the testes, other sections of which are shown in figures b, c) and different stages of testes maturation: **b** mature testis full of spermatozoa (*sz*), **c** immature testis with thick germinative epithelium (*gep*) and narrow lumen (*lum*), **d** testes with both mature and immature lobes; mature lobes are full of spermatozoa (*sz*) and protrude into the peribranchial cavity (*pbc*), whereas the immature lobes have a narrow lumen (*lum*) and are submerged into the trunk coelom (*tc*) of the genital wing, **e** the postspawning testis with a wide empty lumen (*lum*). *ep*, ectodermal epithelium; *gep*, germinative epithelium; *gnp*, gonad pore; *hs*, haemal sinus; *lum*, lumen of the testis; *mc*, muscle cells; *pbc*, peribranchial cavity; *sz*, spermatozoa; *tc*, trunk coelom; *tes*, mature testes; tes*, immature testes

Each testis is a sac with one gonad pore, which opens to the peribranchial cavity (Fig. 4a–c, *gnp*, *pbc*). In the specimens collected in 2018, the genital wings contained mature and immature testes (Fig. 4a, *tes*, tes*) (Table 2). Some testes contain mature and immature lobes at the same time (Fig. 4d). The genital wings of the specimens collected in 2016 were occupied by only lobed testes, which look like in the postspawning stage. These testes contain wide lumen, possess both thin and thick areas of germinative epithelium, and lack spermatozoa (Fig. 4e).

**Table 2:** Characteristics of immature and mature testes in *Quatuoralisia malakhovi*

Maturity of testes	Form of testes	Thickness of germinative epithelium	Lumen	Reference to figures
Immature testes	Strongly lobed	60–100 μm	Narrow, almost empty	3d, 4a,c (tes*), 5d,e
Mature testes	Slightly lobed or pouch-like	30–50 μm	Wide and full of sperm	3c, 4a,d (*tes*), 5a

**Fig. 5:**
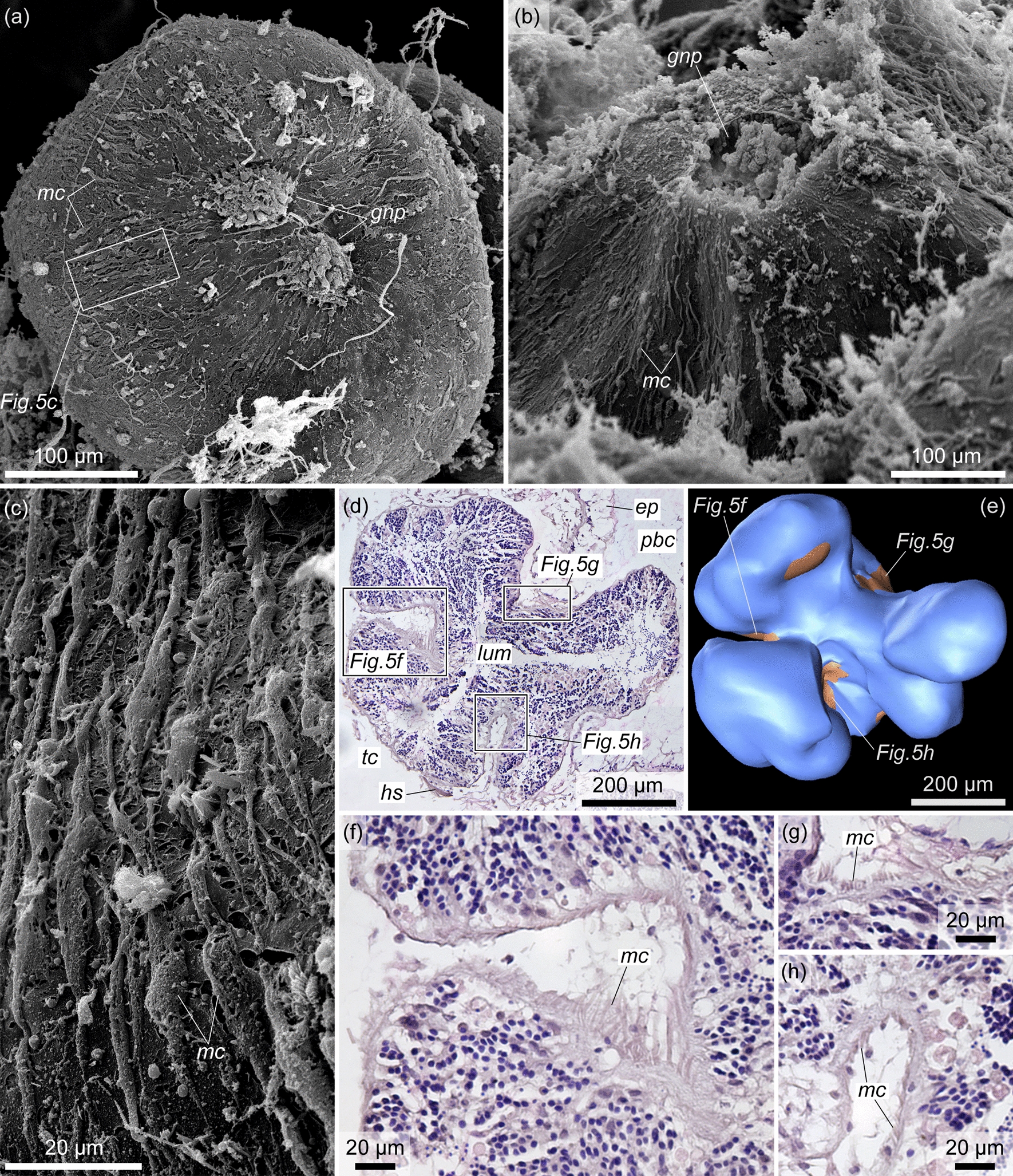
The external muscle lining of the testis in *Quatuoralisia malakhovi*. **a** The whole testis covered by muscle cells (*mc*); the frame indicates the area enlarged in figure c. **b** Gonad pore (*gnp*) surrounded by radial muscle cells. **c** Muscle cells (*mc*) of the external lining of the testis. **d** Contracted muscle cells at the bases of the testis lobes; frames indicate the areas enlarged in figures f–h. **e** Reconstruction of the lobed immature testis (blue) with the areas of contracted muscle cells (brown) at the bases of the lobes. (**f**–**h**) Contracted muscle cells (*mc*) at the bases of the testis lobes. *ep*, ectodermal epithelium; *gnp*, gonad pore; *hs*, haemal sinus; *lum*, lumen of the testis; *mc*, muscle cells; *pbc*, peribranchial cavity; *tc*, trunk coelom

**Fig. 6:**
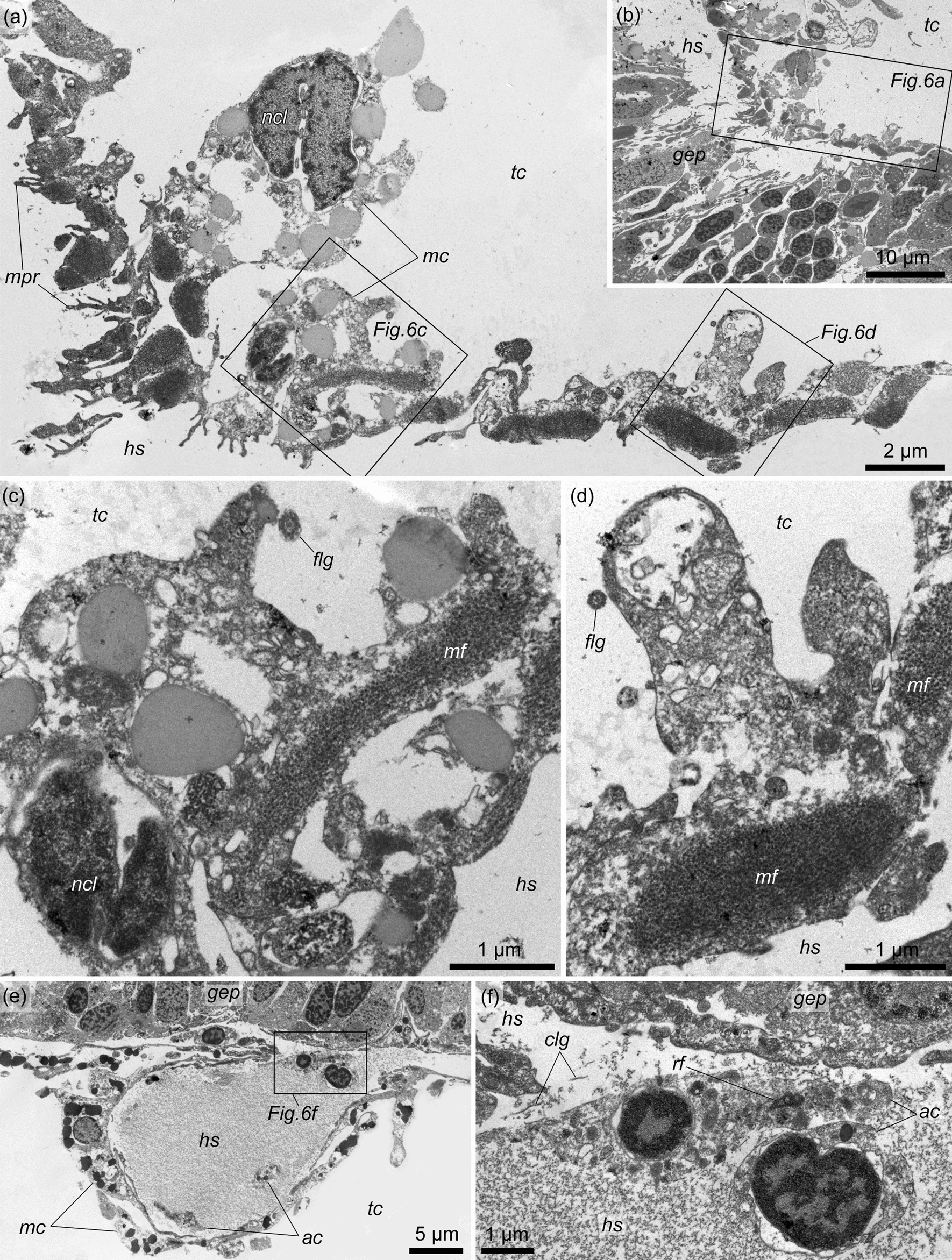
The fine structure of the external wall of the testis in *Quatuoralisia malakhovi*. **a** External muscle lining; frames indicate the areas enlarged in figures c, d. **b** Location of the muscle lining, which envelops the testis; the frame indicates the area enlarged in figure a. **c**–**d** Muscle fibers (*mf*) and cilia (*flg*) of muscle cells. **e** Haemal lacuna (*hs*) of the testis wall; the frame indicates the area enlarged in figure f. **f** Amoebocytes (*ac*) within the haemal lacuna. *ac*, amoebocytes; *clg*, collagen fibers; *flg*, flagellum; *gep*, germinative epithelium; *hs*, haemal sinus; *mc*, nuclear parts of muscle cells; *mf*, muscle fiber; *mpr*, processes of the muscle cells; *ncl*, nucleus; *rf*, rootlet of flagellum; *tc*, trunk coelom

The cellular lining of the external wall of the testis is represented by numerous elongate muscle cells (Fig. 5a, c, *mc*). All muscle cells are located in the same direction from the bottom of the testis to its gonad pore. As a result, the gonad pore is surrounded by a ‘corolla’ of radially elongated muscle cells (Fig. 5a, b). The muscle cells are significantly contracted at the bases of the testis lobes (Figs. 4c, 5d–h). Each muscle cell contains a bundle of myofilaments (i.e., one muscle fiber) with a diameter of 0.5–2 μm (Fig. 6a, c, d, *mf*), which occupy the basal part of the cell. The nuclear parts of the cells contain irregularly shaped nuclei and protrude into the cavity of the trunk coelom (Fig. 6a, c). The rare flagella found on the sections indicate that the muscle cells are monociliary (Fig. 6c, d, *flg*). The basal parts of the muscle cells form numerous slender cytoplasmic processes in the direction of the haemocoel of the testis wall (Fig. 6a, b, *mpr*).

The testis wall contains one or two large haemal lacunae (dilations of the haemal sinus), which are usually located on the bottom of the testis (Figs. 4a, c, d, 5d, *hs*). The haemal lacunae are filled with fine flake-like content and collagen fibers with a striation pattern of 35 nm (Fig. 6e, f). Large amoebocytes are found in the lumen of the haemal lacunae; the endothelial lining is absent (Fig. 6e, f). In the cytoplasm of amoebocytes, the basal bodies and striated rootlets of flagella are rarely visible (Fig. 6f, *rf*).

### Germinative epithelium

**Fig. 7:**
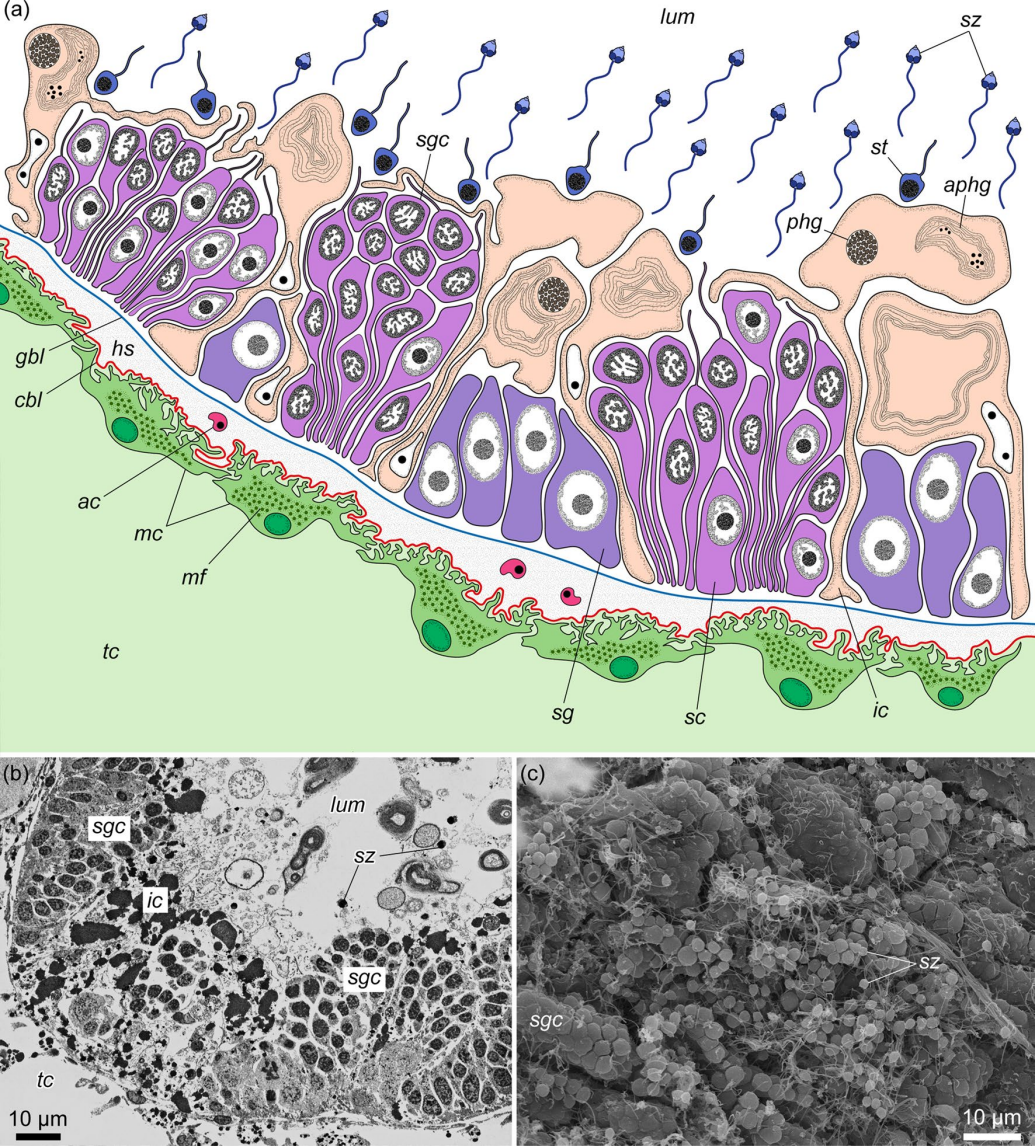
Germinative epithelium of *Quatuoralisia malakhovi*. **a** Diagram. **b** TEM. **c** SEM. *ac*, amoebocyte; *aphg*, autophagosome; *cbl*, coelothelial basal lamina (outer); *gbl*, basal lamina of the germinative epithelium (inner); *hs*, haemal sinus; *ic*, interstitial cell; *lum*, lumen of the testis; *mc*, muscle cell; *mf*, muscle fiber; *phg*, phagosome; *sc*, spermatocyte; *sg*, spermatogonium; *sgc*, spermatogenic column; *st*, spermatids; *sz*, spermatozoa; *tc*, trunk coelom

The germinative epithelium of the testis contains spermatogenic germ cells at different stages of development and somatic interstitial cells (somatic cells). Yolk cells were not found in the testes of *Q. malakhovi*. The spermatogenic cells of various stages are located within the germinative epithelium with radial differentiation (from the testis wall to the lumen) and horizontal compartmentalization (along the testis wall) (Fig. 7). The lower compartments of the spermatogenic cells are 15–20 μm in height and contain predominantly spermatogonia (Fig. 7, *sg*). Relatively tall (40 μm thick) compartments contain spermatogenic columns with spermatocytes at different stages of development (Fig. 7, *sgc*, *sc*). The compartments are separated by interstitial somatic cells (Fig. 7, *ic*).

**Fig. 8:**
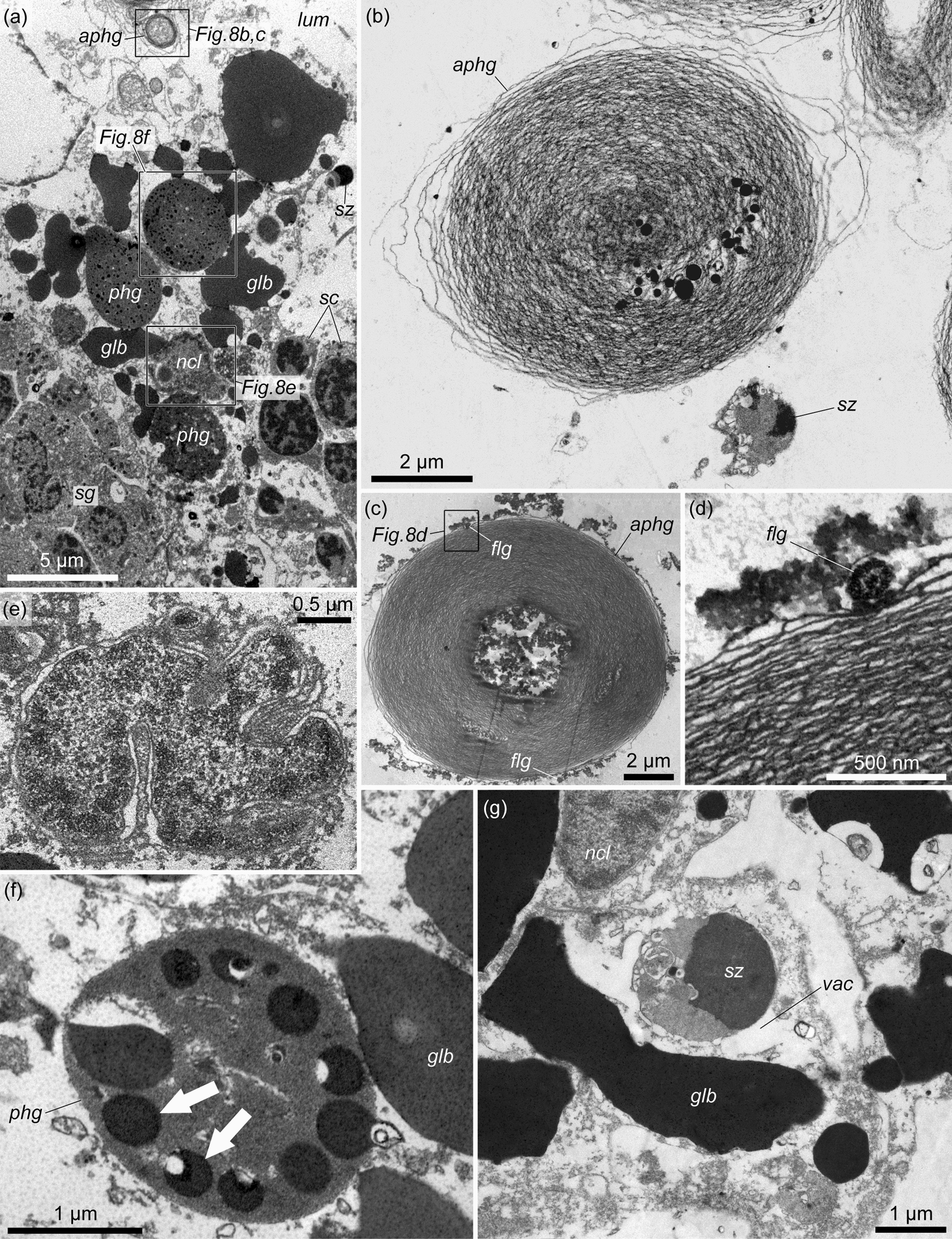
The fine structure of the interstitial cells in the testis germinative epithelium of *Quatuoralisia malakhovi*. **a** General view of the interstitial cells. Frames indicate the structures shown in figures b–f. **b** Autophagosome (*aphg*) and spermatozoon (*sz*) in the cytoplasm of interstitial cell. **c** Autophagosome (*aphg*) and flagella (*flg*); the frame indicates the flagellum enlarged in figure d. **d** Flagellum (*flg*) between the membranes of autophagosome. **e** Nucleus. **f** Phagosome with remnants of degenerating spermatogenic cells (*arrows*). **g** Spermatozoon (*sz*) captured by the interstitial cell. *aphg*, autophagosome; *flg*, flagellum; *glb*, globules; *lum*, lumen of the testis; *ncl*, nucleus; *phg*, phagosome; *sc*, spermatocytes; *sg*, spermatogonia; *sz*, spermatozoon; *vac*, vacuole

Interstitial cells contain light cytoplasm filled with numerous electron-dense globules of various shapes (Fig. 8a, *glb*). The most characteristic feature of interstitial cells is the presence of large, rounded, electronically dense phagosomes (phagocytic vacuoles) containing remnants of degenerating spermatogenic cells (Fig. 8a, f, *phg*) and multimembrane bodies – autophagosomes (Fig. 8a–d, *aphg*). The latter are myelin-like formations, which arise around the captured spermatogenic cells (Fig. 8g). Among the numerous membrane layers of autophagosomes, the remnants of degenerating spermatogenic cells are also visible (Fig. 8b–d). The central area of the autophagosome is often occupied by flake-like or fine-grained material (Fig. 8c). The nuclei of interstitial cells have an irregular shape, often with deep invaginations (Fig. 8e). There are one or two nucleoli. Interstitial cells form numerous processes embracing spermatogenic cells and spermatogenic columns and separating them from each other.

Germ cells of the testes include four different stages of spermatogenesis: spermatogonia, spermatocytes, spermatids, and spermatozoa.

### Spermatogenesis

**Fig. 9:**
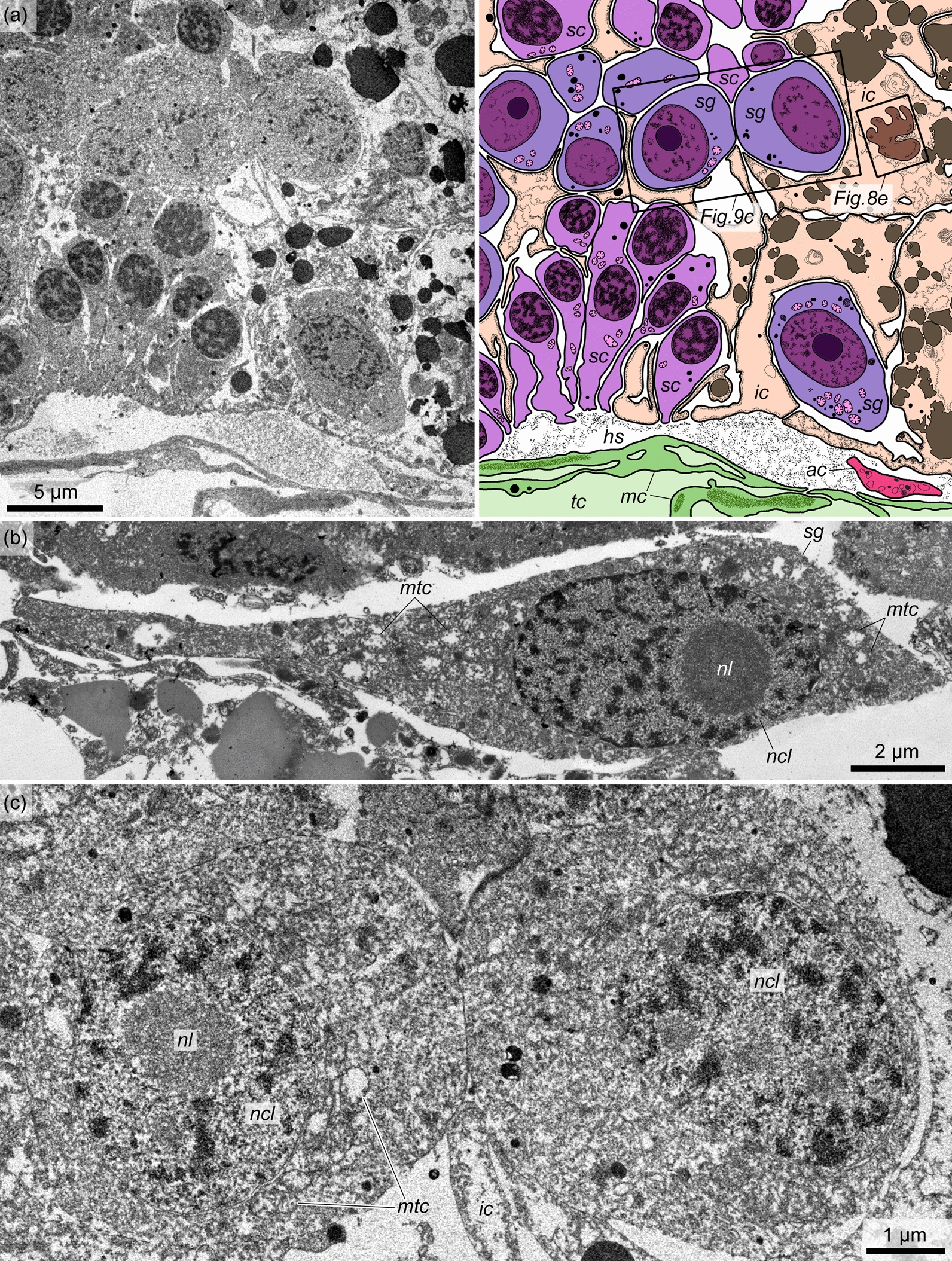
The fine structure of the spermatogonia of *Quatuoralisia malakhovi*. **a** Basal part of the germinative epithelium containing spermatogonia (*sg*), spermatocytes (*sc*), and interstitial cells (*ic*). Frames show the areas enlarged in Fig. 8e and 9c. **b** Longitudinal section of spermatogonium (*sg*). **c** Transverse section of two adjacent spermatogonia. *ac*, amoebocyte; *sc*, spermatocyte; *sg*, spermatogonium; *hs*, haemal sinus; *ic*, interstitial cell; *mc*, muscle cell; *mtc*, mitochondrion; *ncl*, nucleus of spermatogonium; *nl*, nucleolus; *tc*, trunk coelom

*Spermatogonia* are large oval or spindle-shaped cells 20–25 µm high with a diameter of approximately 5–10 µm in the nuclear area (Fig. 9). These cells occupy a basal position in the germinative epithelium, and are often located near the haemal lacunae. Spermatogonia are separated from other spermatogenetic stages by the processes of interstitial cells (Fig. 9a). The nuclei of spermatogonia are large, with a diameter of up to 5–6 µm, with one, rarely two nucleoli with a diameter of 1.5–2 µm (Fig. 9b, c, *ncl*, *nl*). Condensed chromatin is distributed along the periphery of the nucleus and widely scattered over the central area so that the nucleus looks very light. The cytoplasm of spermatogonia contains oval mitochondria of different sizes that are evenly distributed throughout the cell, a few small electron-dense granules, a small number of electron-transparent vesicles, and the Golgi apparatus. Smooth endoplasmic reticulum (SER) tubules are found both in the near-nuclear area and on the periphery of the cell. We did not find flagella in the spermatogonia. Spermatogonia divide by mitosis, giving rise to spermatocytes.

**Fig. 10:**
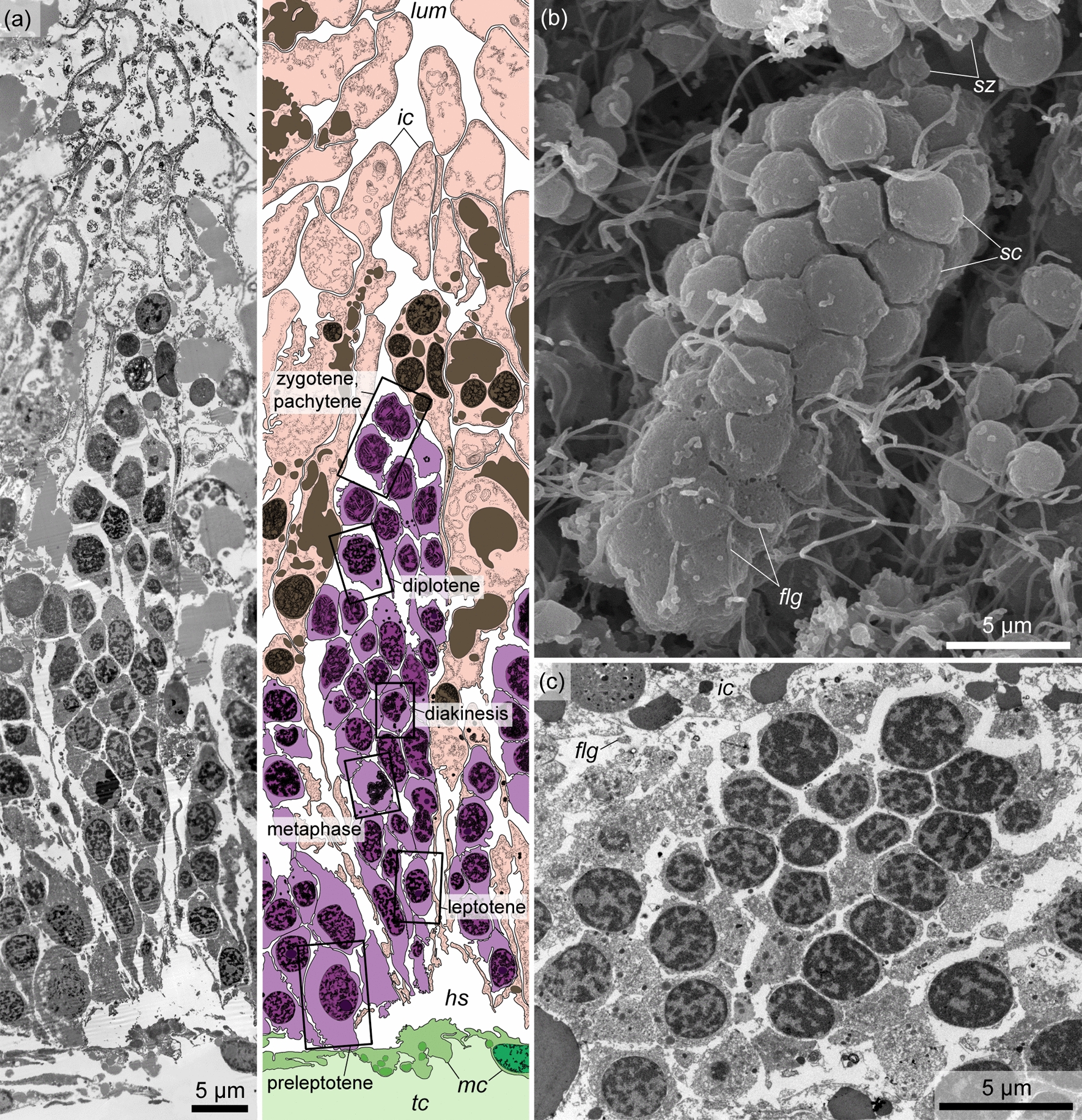
Spermatogenic column in the germinative epithelium of *Quatuoralisia malakhovi*. **a** General view of the spermatogenic column containing different stages of primary spermatocytes (frames), and enveloped by interstitial cells. **b** SEM image of the spermatogenic column enveloped in the basal part by thin interstitial cells penetrated by flagella (*flg*). **c** TEM image of spermatocytes packed to the spermatogenic column. *flg*, flagellum; *hs*, haemal sinus; *ic*, interstitial cell; *lum*, lumen of the testis; *mc*, muscle cell; *sc*, spermatocyte; *sz*, spermatozoa; *tc*, trunk coelom

**Fig. 11:**
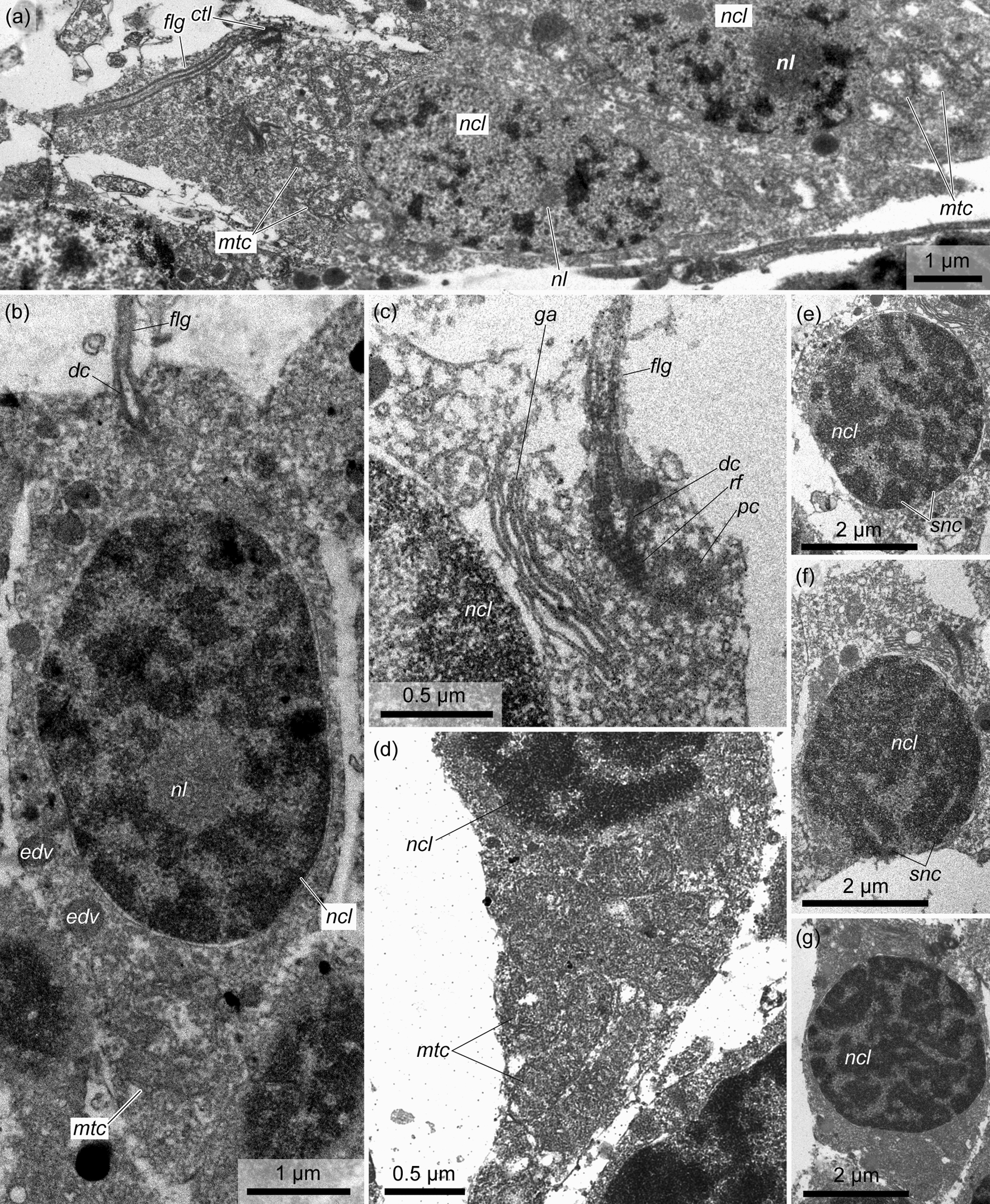
Fine structure of primary spermatocytes in the germinative epithelium of *Quatuoralisia malakhovi*. **a** Preleptotene spermatocyte. **b** Leptotene spermatocyte. **c** Apical area of the zygotene spermatocyte. **d** Basal part of a pachytene spermatocyte. **e** Nucleus of a zygotene spermatocyte. **f** Nucleus of a pachytene spermatocyte. **g** Nucleus of a diplotene spermatocyte. *ctl*, centriole; *dc*, distal centriole; *edv*, electron dense vesicle; *flg*, flagellum; *ga*, Golgi apparatus; *mtc*, mitochondrion; *ncl*, nucleus; *nl*, nucleolus; *pc*, proximal centriole; *rf*, rootlet filament; *snc*, synaptonemal complex

**Fig. 12:**
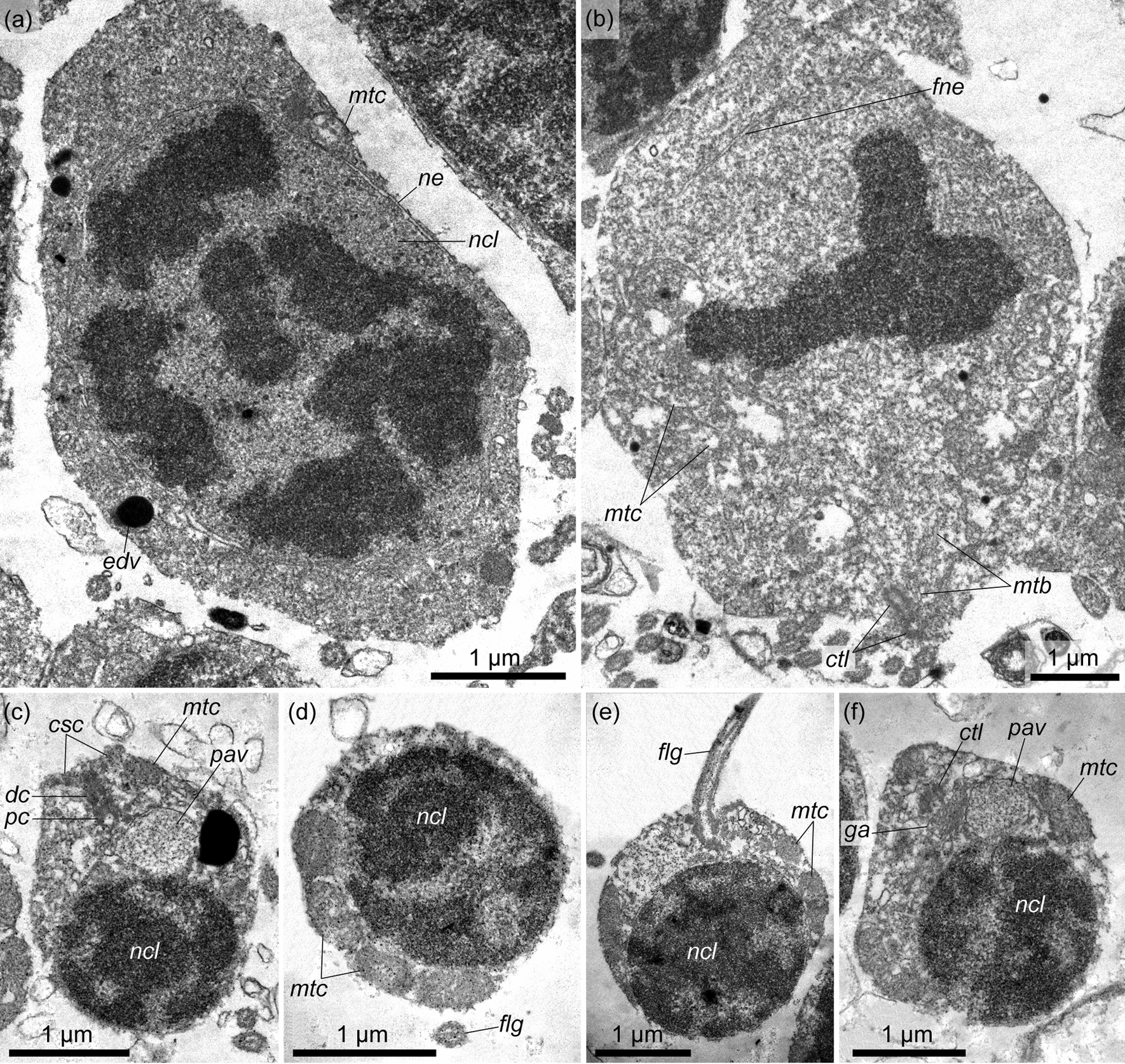
Fine structure of diakinesis spermatocyte **a**, metaphase I spermatocyte **b**, and spermatids in the testes of *Quatuoralisia malakhovi*: **c** spermatid basal apparatus, **d** mitochondria in the spermatid, **e** spermatid flagellum, **f** Golgi apparatus near the basal apparatus of the spermatid. *csc*, centriolar satellite connectives; *ctl*, centriole; *dc*, distal centriole; *edv*, electron dense vesicle; *flg*, flagellum; *fne*, fragmented nuclear envelope; *ga*, Golgi apparatus; *mtb*, microtubules; *mtc*, mitochondrion; *ncl*, nucleus; *ne*, nuclear envelope; *pav*, proacrosomal vesicle; *pc*, proximal centriole

*Spermatocytes* are found throughout the total thickness of the germinative epithelium of the testis, from its wall to the lumen. These cells are clustered and thus form spermatogenic columns (Figs. 7, 10, *sgc*). Adjacent spermatogenic columns are separated from each other by interstitial cells, which form a thin envelope around the spermatogenic column and are pierced by the flagella of spermatocytes (Fig. 10, *ic*). The nuclei of spermatocytes are 3–4 µm in diameter (Fig. 11a, b, e–g, *ncl*), and their chromatin is more condensed than that of spermatogonia. The Golgi apparatus is located in the vicinity of the nucleus (Fig. 11c, *ga*), and the latter is surrounded by numerous oval mitochondria (Fig. 11a, b, d, *mtc*). The preleptotene spermatocytes occupy a near-wall position in the germinative epithelium and have a very similar appearance to that of the spermatogonia, i.e., they are large spindle-shaped cells with a large nucleus containing more condensed chromatin distributed throughout the nucleus in small, frequent spots and a nucleolus 1–1.5 µm in diameter (Fig. 11a, *ncl*, *nl*). In some preleptotene spermatocytes, a flagellum and its basal apparatus immersed in the cytoplasm are found (Fig. 11a, *flg*). The flagellum of spermatocytes from the leptotene to diplotene stages is not immersed in the cytoplasm but freely rises above the cell (Fig. 11b, c, *flg*). Its basal apparatus is in close proximity to the Golgi apparatus and includes two centrioles and a striated rootlet (Fig. 11c). Zygotene spermatocytes and pachytene spermatocytes are determined by the synaptonemal complex in the nucleus (Fig. 11e, f, *snc*). In diplotene spermatocytes, the condensed chromatin forms ring-shaped structures (Fig. 11g). At the stage of diakinesis, the primary spermatocytes are already devoid of flagellum and rootlet filaments (Fig. 12a). The nuclear envelope is fragmented, and the chromatin in the nucleus is located in several large spots. In metaphase spermatocytes, the nuclear envelope is highly fragmented. The condensed chromatin forms a single ribbon across the cell, and on the sides of it, the centrioles and microtubules extending from them are found in the periphery (Fig. 12b).

*Spermatids* are small cells (2–2.2 µm in diameter) with a high nuclear–cytoplasmic ratio (Fig. 12c–f). They are formed as a result of the second division of meiosis and occupy the space in the periphery of the testis lumen. The diameter of the spermatid nuclei is 1.5–2 µm. The spermatid cytoplasm contains the proacrosomal vesicles produced by the Golgi apparatus (Fig. 12c, f, *pav*, *ga*), and the mitochondria accumulate in the area between the nuclei and centrioles of the flagellum (Fig. 12d–f). The centriolar satellite connectives extend from the distal centriole of the flagellum (Fig. 12c, *csc*). During spermiogenesis, chromatin in the nuclei of spermatids condenses, proacrosomal vesicles migrate to the apical part of the cell, and as a result, spermatozoa are formed.

**Fig. 13:**
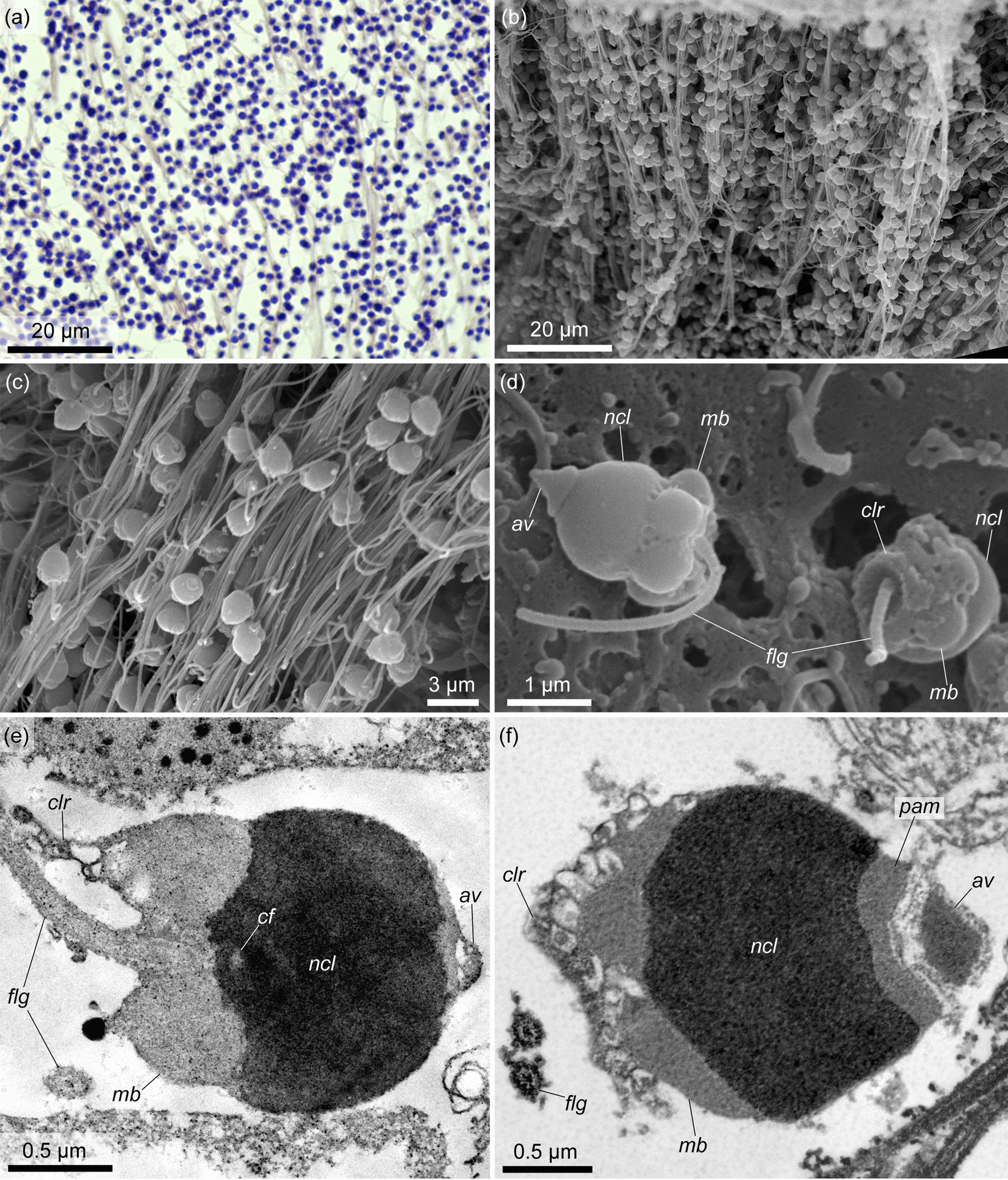
Fine structure of the spermatozoa of *Quatuoralisia malakhovi*. **a** Spermatozoa in the histological section. **b** Bundles of spermatozoa in the testis cavity (SEM). **c** Spermatozoa united into bundles. **d** External structure of spermatozoa. **e**, **f** Internal structure of spermatozoa. *av*, acrosomal vesicle; *cf*, centriolar fossa; *clr*, cytoplasmic collarette; *flg*, flagellum; *mb*, mitochondrial body; *ncl*, nucleus; *pam*, periacrosomal material

*Spermatozoa* accumulate in the lumen of the testis. In mature testes, they occupy almost the entire testis lumen (Fig. 4a, d). Mature spermatozoa are united into dense bundles, which are probably formed due to the adhesive properties of spermatozoon flagella (Fig. 13a–c). The bundles of spermatozoa are parallel to each other and run radially from the wall of the testis to its lumen. A spermatozoon consists of an acorn-shaped elongated head 2 µm in length and a flagellum 18–25 µm long (Fig. 13c, d). The head includes a distinguishable beak-shaped acrosomal complex, a spherical nucleus and a midpiece with a ring of 5 or rarely 6 mitochondria (Fig. 13d–f).

The acrosomal complex is slightly immersed into the small fossa at the anterior end of the nucleus. The acrosomal vesicle contains an electron-dense fine-grained material; it is separated from the nucleus by the layer of the periacrosomal material of a diffuse structure (Fig. 13f, *av*, *pam*). The strongly condensed nucleus is 1.1 µm long and 1.5–1.8 µm wide. The posterior part of the nucleus is depressed by the mitochondria and in the area of the centriolar fossa (Fig. 13e, *cf*). The flagellum extends from the center of the posterior area of the sperm cell. A small cytoplasmic collarette surrounds the base of the flagellum (Fig. 13d-f, *clr*).

## Discussion

The bulging of numerous testes into the peribranchial cavity above the genital wing surface is one of the specific features of *Q. malakhovi* [5]. Such protruding testes were not described either for enteropneusts or for hemichordates in general. In other acorn worms, the testes are entirely embedded into the body within the genital wings or genital ridges [6, 8, 11, 13, 14, 33–35]. However, in *Q. malakhovi*, the bulging gonadal structures are only the lobes of the testes, one or several from each testis. The central part of the testis is always submerged in the thickness of the genital wing and the gonad pore opens in this central part, not in the bulging lobes (Fig. 4a–c). The protrusion of the testis lobes may be a temporary phenomenon caused by the maturation of the testis, which becomes filled with spermatozoa so much that some lobes are extruded into the peribranchial cavity. On the other hand, in other acorn worms, mature and filled of sperm testes are embedded into the body and do not protrude [6, 23, 33, 34]. The muscle cells stretch along the testis from the bottom to the gonad pore surrounding the latter (Fig. 5a–c), and this arrangement, as well as the contraction of the muscle cells at the bases of the testis lobes (Fig. 5d–h) suggests that the testes are squeezed by the muscle cells for spawning. The synchronous contraction of the muscle lining probably results in the withdrawal of sperm from the lobes into the main lumen of the testis and then through the gonad pore into the peribranchial cavity.

One more similarity between the torquaratorids and harrimaniids (except *Saccoglossus*) is the absence of yolk cells in the testes [23]. Thus, the nutritional role in the testes of *Q. malakhovi* involves the haemal lacunae and interstitial cells. The spermatogonial cells and primary spermatocytes of the spermatogenic columns connect with the basal lamina of the germinative epithelium and can obtain nutrients directly from the blood (Figs. 6e, f, 7a, 9a, 10a). The nutrition of secondary spermatocytes and spermatids is likely provided by interstitial cells, which retain their connection with the basal lamina of the germinative epithelium. In addition, the interstitial cells contain many electron-dense globules (Fig. 8a, g) and phagocytize degenerating spermatogenic cells and residual cytoplasm, which is discarded during the formation of spermatozoa (Fig. 8b–d, f, g). It is possible that interstitial cells also supply spermatogonia and primary spermatocytes.

There are very few studies on the development and structure of the spermatozoa of Enteropneusta, but together, they are meaningful since they cover three of the four families of the class: Harrimaniidae, Spengelidae, and Ptychoderidae [33, 36–41]. A detailed comparative analysis of the spermatozoa of representatives of these families was performed by Jamieson [25] and Franzén [41]. Our study of *Q. malakhovi* adds information on the family Torquaratoridae and makes it possible to improve conclusions previously made.

Common to all four families is the so-called primitive sperm [40, 42] or ectaquasperm [43] with a head and flagellum. The head contains a dense rounded nucleus with a frontal acrosomal vesicle separated from the nucleus by periacrosomal material. In the spermatozoon midpiece, a ring of mitochondria surrounds a depression with a pair of centrioles anchored by the fiber apparatus. A flagellum has the usual 9 + 2 axoneme starting from the distal centriole. There are differences between the spermatozoa of the four families related only to the details of the described basic pattern and they may have phylogenetic significance within enteropneusts.

The sperm heads of *Saccoglossus kowalevsky* (Harrimaniidae), *Schizocardium* sp. (Spengelidae), and *Ptychodera flava* (Ptychoderidae) are described as roundish, in *Balanoglossus carnosus* (Ptychoderidae) and *Sac. horsti* (Harrimaniidae) as spherical, in *Saxipendium coronatum* (Harrimaniidae) as pyramidal [22, 23, 33, 36, 38–41], and as acorn-shaped in *Q. malakhovi*. Nuclei and acrosomes also vary in shape and structure. *Sax. coronatum* has four characteristic frontal ridges radiating from the large acrosome. The acrosomes are described as dome-shaped in *Sac. kowalevskii*, as small, rounded or pointed in *B. carnosus* and *Sac. horsti*, and as beak-shaped in *Q. malakhovi*.

In all the species studied, the sperm midpiece contains a ring of spherical or flattened mitochondria, but the structure of this ring is described in different ways. 4–5 mitochondria are mentioned for *Sax. coronatum* sperm, but it is assumed that they merge into one annular mitochondrion [33]. A single mitochondrion representing a large mitochondrial ring has been described for the sperm of *Glossobalanus minutus* (Ptychoderidae) [24]. In *Schizocardium* sp. sperm, the midpiece contains four spherical mitochondria [41]. Four mitochondria have also been described for *B. carnosus*, *P. flava*, *Sac. kowalevskii*, and *Sac. horsti* sperm [22, 23, 36, 37]. In *Q. malakhovi*, the number of mitochondria in the midpiece varies, and 5 or rarely, 6 mitochondria can be distinguished on SEM images.

Thus, enteropneusts differ in the shape of the sperm head and acrosome, as well as in the number and structure of the mitochondria of the sperm midpiece. Without studying the spermatozoa of other genera of these four families, phylogenetic conclusions about the specificity of sperm morphology to each family can be preliminary. Notably, in *Q. malakhovi*, a representative of the family of clearly deviant deep-sea enteropneusts with specific lifestyle, spermatogenesis and spermatozoa generally follow the pattern of the other three families.

The clear similarity of the spermatozoa of enteropneusts and nonechinoid eleutherozoan echinoderms (Asteroidea, Ophiuroidea, and Holothuroidea) may reflect the close phylogenetic relationships of these two phyla of Deuterostomia [25, 41, 44]. This looks logical from the point of view of a formal morphological analysis; however, the authors mention that primitive spermatozoa, by definition, possess a set of plesiomorphic features and themselves represent a plesiomorphic trait in the metazoan life cycle. Spermatozoa, called primitive or ectaquasperm, are well known in a variety of nonrelated aquatic invertebrates of Bilateria and even Radiata [41, 45]. The primitive spermatozoa as a plesiomorphic trait are poorly informative for the phylogeny of higher taxa. At the same time, noticeable deviations from the primitive pattern (for example, annular mitochondria in sea urchin sperm) are significant for phylogenetic conclusions [41].

## Conclusions

The first detailed description of the torquaratorid male reproductive system and sperm structure is provided. In *Q. malakhovi*, the testes and spermatozoa have a number of differences from other studied acorn worms, namely, the bulging of the mature lobes of the testes into the peribranchial cavity, the muscle cells ‘corolla’ surrounding the gonad pore, the beak-shaped acrosomal part of the sperm head, and 5–6 mitochondria in the spermatozoon midpiece. However, in general, the male system, spermatogenesis, and spermatozoa structure of the torquaratorid *Q. malakhovi* follow the pattern of the other three enteropneust families, and the phylogenetic significance of the identified deviations should be the subject of further research.

## Data Availability

The datasets used and/or analyzed during the current study are available from the corresponding author upon reasonable request.
